# Proto-oncogene c-erbB2 initiates rat primordial follicle growth via PKC and MAPK pathways

**DOI:** 10.1186/1477-7827-8-66

**Published:** 2010-06-19

**Authors:** Zheng Li-Ping, Zhang Da-Lei, Huang Jian, Xu Liang-Quan, Xu Ai-Xia, Du Xiao-Yu, Tang Dan-Feng, Zheng Yue-Hui

**Affiliations:** 1 Department of Physiology Reproduction, Medical College of Nanchang University, Nanchang, China

## Abstract

**Background:**

c-erbB2, a proto-oncogene coding epidermal growth factor receptor-like receptor, also as a chemosensitivity/prognosis marker for gynecologic cancer, may be involved in initiation of growth of rat primordial follicles. The aim of the present study is to investigate the role and signal pathway of c-erbB2 in onset of rat primordial follicle development.

**Methods:**

The expression of c-erbB2 mRNA and protein in neonatal ovaries cultured 4 and 8 days with/without epidermal growth factor (EGF) were examined by in situ hybridization, RT-PCR and western blot. The function of c-erbB2 in the primordial folliculogenesis was abolished by small interfering RNA transfection. Furthermore, MAPK inhibitor PD98059 and PKC inhibitor calphostin were used to explore the possible signaling pathway of c-erbB2 in primordial folliculogenesis.

**Results:**

The results showed that c-erbB2 mRNA was expressed in ooplasm and the expression of c-erbB2 decreased after transfection with c-erbB2 siRNA. Treatment with EGF at 50 ng/ml significantly increased c-erbB2 expression and primary and secondary follicle formation in ovaries. However, this augmenting effect was remarkably inhibited by c-erbB2 siRNA transfection. Furthermore, folliculogenesis offset was blocked by calphostin (5 × 10(-4) mmol/L) and PD98059 (5 × 10(-2) mmol/L), but both did not down-regulate c-erbB2 expression. In contrast, the expressions of p-ERK and p-PKC were decreased obviously by c-erbB2 siRNA transfection.

**Conclusions:**

c-erbB2 initiates rat primordial follicle growth via PKC and MAPK pathways, suggesting an important role of c-erbB2 in rat primordial follicle initiation and development.

## Background

Folliculogenesis is a complex process consisting of sequential and ordered follicular development and growth. Although much is known about the events and regulation of the later stages of ovarian follicular development, the early follicular development is very poorly understood. More recently, attention has focused on regulation of the initiation of follicular growth (follicle activation) [1,2]. The initiation of follicular growth and progression beyond the primary follicle stage requires locally produced factors and peptides, which can occur without gonadotrophins [3-6]. The local growth factors such as epidermal growth factor (EGF), stem cell factor (SCF), basic fibroblast growth factor (bFGF) and serum anti-Mullerian have been known to be necessary to induce primordial follicle development and initiate folliculogenesis [7-12].

There is accumulating evidence implicating EGF as a key regulator of primordial follicle development in mammals. EGF has been shown, as mitogen for cultured granulosa cells, to stimulate oocyte growth during the primordial to primary follicle transition in vitro [8,13], and EGF receptor has been demonstrated in oocytes from primordial and primary follicles in many kind of species [14-16]. Furthermore, EGF triggered primordial follicle development by stimulating proliferation of granulosa cells [17]. However, the molecular mechanism by which EGF triggers primordial follicle development has not been fully clarified. *c-erbB*_*2*_, a member of the EGF receptor family, encoding a transmembrane EGF receptor [18,19], is expressed in primordial germ cells, granulosa cells, luteal cells and oocytes [20,21]. *c-erbB*_*2 *_is also reported as a marker for chemosensitivity and prognosis of breast and ovarian cancer [22,23].

Recently, we focused on characterizing the effect of *c-erbB*_*2 *_on oocyte maturation and found that *c-erbB*_*2 *_induced oocyte maturation via activation of mitogen-activated protein kinase (MAPK) [24]. MAPKs are known as extracellular-signal-regulated kinases (ERKs), and ERK1/2 are known as classical MAP kinases. In the present study, we tested the expression of *c-erbB*_*2 *_mRNA and protein translation and investigated the role and signaling pathway of *c-erbB*_*2 *_in primordial follicle development. In addition, we explore the molecular mechanism of EGF effect on primordial folliculogenesis.

## Methods

### Animals and reagents

Animal use was approved by the Committee of Nanchang University for Animal Research. Sprague Dawley rats (obtained from the Animal Care of Medical College of Nanchang University) were used for all the experiments. EGF, PD98059 (a MAPK inhibitor) and calphostin (a PKC inhibitor) were purchased from Sigma (St. Louis MO).

### Histology and organ culture

Ovaries from 2-day-old rats were collected fresh or cultured for 4 and 8 days, with 20 ovaries in each group. Fresh ovaries were fixed in Bouins solution for 1-2 h, embedded in paraffin, sectioned (3-5 × 10^-3 ^mm), and were stained with hematoxylin and eosin. The number of follicles at each developmental stage was counted in two serial sections from the largest cross-section through the center of the ovary [25]. Normally, two ovaries were in each treatment group as a replicate and 150-200 follicles were present in an ovary cross-section. Experiments were repeated three times (therefore, n = 6 for each treatment group). Follicles were classified as either primordial (stage 0), or as one of the developing preantral stages (stage 1-4) as described previously [25]. Briefly, primordial follicles consist of one oocyte partially or completely encapsulated by flattened squamous pregranulosa cells. Developing (stage 1-4) follicles contain successively more cuboidal granulosa cells in layers around the oocyte. Whole ovaries were cultured on sponge in 0.5 ml of Waymouth MB 752/1 medium supplemented with 0.1% BSA (Sigma, St. Louis, MO), 0.1% albumax (Gibco BRL, Gaithersburg, MD). Ovaries were cultured at 37°C with 5% CO_2 _in 4-well plates (Nunc plate; Applied Scientific, South San Francisco, CA), ovaries were randomly assigned to treatment groups with 1-3 ovaries per well. The medium was changed every 2 days. During organ culture, ovaries were treated with EGF (50 ng/ml) and *c-erbB*_*2 *_small interfering RNA (siRNA, 0.1 mmol/L) alone or in combinations. In addition, ovaries were challenged with PD98059 (5 × 10^-2 ^mmol/L) or calphostin (5 × 10^-4 ^mmol/L).

### In situ hybridization

Localization of *c-erbB*_*2 *_mRNA was determined by in situ hybridization. The *c-erbB*_*2 *_multiphase oligonucleotide probes digoxigenin-labeled were as follows: 1)5'-G G A A GGACGTCTTCCGCAAGAATAACCAACTGGCT-3';2)5'-G C T T T G T A C A C ACTGTACCTTGGGACCAGCTCTTC-3';3)5'-C G G A C C T C T C C T A C A T G C CCATCTGGAAGTACCCG3'.Before hybridization, the liquids and containers have been strictly treated with 0.1% DEPC. Slides were deparaffinized and rehydrated with 3% H_2_O_2_, and subjected to enzymatic digestion with pepsin for 2-3 min(diluted with 3% citric acid, fresh), and then incubated in pre-hybridization solution at 37°C for 2 h. After discarding the prehybridization solution, the slides were transferred to hybridization solution overnight with water, covering the specimens on special coverslips of the situ hybridization. The next morning the coverslips were opened and washed three times in 2 × SSC (standard saline Citrate, containing Nacl 3 mol/L, sodium citrate 0.3 mol/L), 0.5 × SSC, 0.2 × SSC, and then were incubated with the incubating solution at 37°C for 30 min. Slides were exposed to biotinylated mouse anti-digoxigenin IgG for 60 min. Finally, the immunoreactions were detected by using SABC (Strept-Avidin-Biotin Complex) system. Slides were counterstained with haematoxylin before observation. As negative control we used pre-hybridization solution which without probe to replace hybridization solution with probe solution.

### Quantitative reverse transcriptase-polymerase chain reaction (RT-PCR)

Expression of mRNA for *c-erbB*_*2 *_was assayed by RT-PCR. Ovaries from the same culture well were pooled to make single RNA sample. RNA was extracted using the Trizol reagent (Sigma, St. Louis, MO). Total RNA from each sample was reverse transcribed into cDNA using a standard oligo-dT RT protocol. cDNA samples were used as template for polymerase chain reaction (PCR) analysis. The 2 × EasyTaq PCR Supermix kit (TRansGen Biotec) was used according to the manufacturer's instructions. The *c-erbB*_*2 *_primers forward: 5'-CAGTGTGTCAACTGCAGTCA-3', reverse: 5'-CAGGAGTGGGTGCAGTTGAT-3'. The housekeeping reference gene GAPDH primers forward: 5'-GCAAGTTCAACGGCACAG-3', reverse:5'-AGGTGG AAG AATGGGAGTTGCT- 3'. The protocol was 94°C for 4 min, then 35 cycles of 95°C for 15 sec, 55°C for 60 sec and 72°C for 2 min. Fluorescent detection data were analyzed and normalized for *c-erbB*_*2 *_mRNA levels to GAPDH mRNA levels. The identities of the PCR products were confirmed by direct sequencing (Shanghai Sangon Biological Engineering Technology & Services Co., Ltd)

### Western blotting analysis

Tissue protein extracts were electrophoretically separated under reduced conditions using NuPAGE 4-12% Bis-Tris gels (Invitrogen; Paisley, UK). Standard Mark (Invitrogen) was used as the molecular weight standard. Proteins were then electrotransferred to nitrocellulose membranes (BIORAD; Munich, Germany) and the immunoblots were subsequently blocked for 2 h at room temperature in TBST (TBS containing 0.1% Tween 20) containing 5% nonfat dry milk. The membranes were incubated overnight at 4°C with antibodies against PCNA, ErbB_2_, p-ERK1/2, p-PKC or β-actin (Sigma). The β-actin bands were used as an internal control for equal loading. After rinsing with TBST, the membranes were incubated for 30 min at room temperature with horseradish peroxidase-conjugated anti-rabbit or anti-mouse secondary antibodies (Amersham; Aylesbury, UK). Finally, the membranes were stained with DAB according to the manufacturer's instructions and analyzed with Gel image analysis system.

### Designing and transfecting of c-erbB_2 _siRNA

EASY siRNA kit was purchased from Shanghai Chemical Technology Co., Ltd. target to *c-erbB*_*2 *_gene (NM_017003). The *c-erbB*_*2 *_siRNA was as follows: sense, 5'-CAGUACCUUCUACCGUUCAtt-3', antisence, 5'-UGAACGGUAGAAGGUA CUGtt-3'. Transfection was followed on the manufacturer's instructions. Briefly, 3 × 10^-3 ^ml 2 × 10^-2 ^mM of siRNA and 2 × 10^-3 ^ml of liposomes (METAFECTENE) were each added to in 5 × 10^-2 ^ml free of serum and antibiotics medium respectively, and the two solutions were combined without any mixture procedures and incubated at room temperature for 15-20 min. After incubation, siRNA-lipid complexes were added to culture flasks (containing 0.5 ml medium and ovaries) and swirl flasks and incubated at 37°C in CO_2 _incubator. The final siRNA concentration of transfection was 0.1 mmol/L. Ovaries were cultured with/without 0.1 mmol/L targeting siRNA for 12 h. The medium was replaced after 12 h transfection with fresh medium containing no siRNA, and ovaries were cultured for 24 h and then collected to detect gene expression and protein translation by using RT-PCR and western-blot. Ovaries without transfection were used as the control. The negative control was the group transfected with negative siRNA. In addition, ovaries were processed for morphometric assessment of the development of primordial follicles.

### Statistics

The experiment was repeated three times. All data were presented as the means ± SEM and analyzed by ANOVA and Duncan's new multiple range tests. *p *< 0.05 was considered significantly difference.

## Results

### Expression of c-erbB_2 _in the ovaries during the initiation of growth of primordial follicle

**Figure 1:**
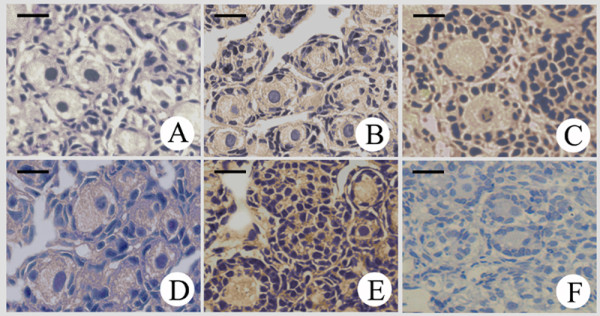
**In situ hybridization detection of *c-erbB***_***2***_** mRNA in the ovaries**. (A), Control (ovary of 2-day-old rat); (B), Ovary of 2-day-old rat cultured for 4 days; (C), Ovary of 2-day-old rat cultured for 8 days; (D), Ovary of 2-day-old rat cultured for 4 days with 50 ng/ml EGF; (E), Ovary of 2-day-old rat cultured for 8 days with 50 ng/ml EGF; (F), Negative control. Scale bar: 2.5 × 10^-2 ^mm.

To examine the expression of *c-erbB*_*2*_, in situ hybridization and RT-PCR were performed. Hybridization histochemistry demonstrated that *c-erbB*_*2 *_mRNA was expressed in ooplasm from primordial follicles of 2 day postnatal ovaries to cultured 8 days of ovaries. Moreover, *c-erbB*_*2 *_mRNA expression increased with prolonged culture, especially in proliferating cumulus cells of cultured ovaries. To investigate more direct actions of EGF, ovaries were incubated in the absence or presence of EGF before RNA collection and analysis. After treatment with EGF (50 ng/ml), the ovaries showed more intense labeling for *c-erbB*_*2 *_mRNA than the control (Fig. 1).

**Figure 2:**
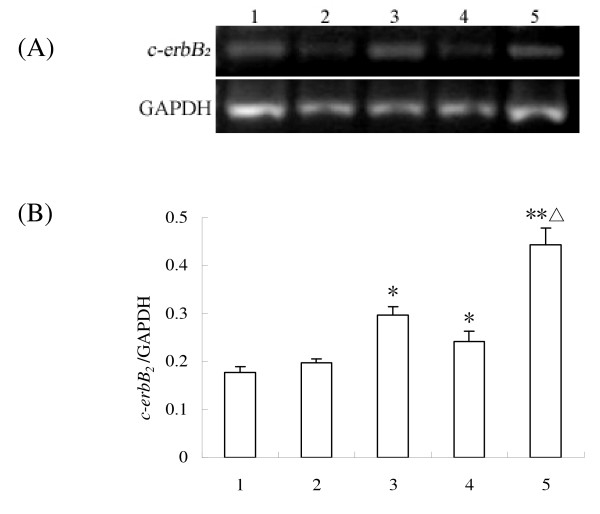
**RT-PCR detection of *c-erbB***_***2***_** mRNA in the ovaries**. (A), The expressions of *c-erbB*_*2 *_mRNA (322 bp) by RT-PCR; (B), Semiquantitative analysis of the RT-PCR result; (1), Control (ovary of 2-day-old rat); (2), Ovary of 2-day-old rat cultured for 4 days; (3), Ovary of 2-day-old rat cultured for 8 days; (4), Ovary of 2-day-old rat cultured for 4 days with 50 ng/ml EGF; (5), Ovary of 2-day-old rat cultured for 8 days with 50 ng/ml EGF. Data are presented as means ± SEM (n = 3). *, P < 0.05; **, P < 0.01 vs control group; Δ, P < 0.05 vs group 3.

After RT-PCR, cDNA was amplified from RNA extracted from cultured ovaries to assess *c-erbB*_*2 *_expression in response to different treatments (Fig. 2). As previously discussed, the neonatal ovary is primarily composed of early-stage primordial follicles, which can initiate growth when cultured in vitro. As shown in Fig. 2, *c-erbB*_*2 *_mRNA appeared more abundant with primordial follicles that had initiated growth and EGF treatment increased *c-erbB*_*2 *_mRNA expression compared to controls (*P *< 0.05), and the results were consistent with in situ hybridization analysis.

### Western blotting detection of PCNA protein in the ovaries

**Figure 3:**
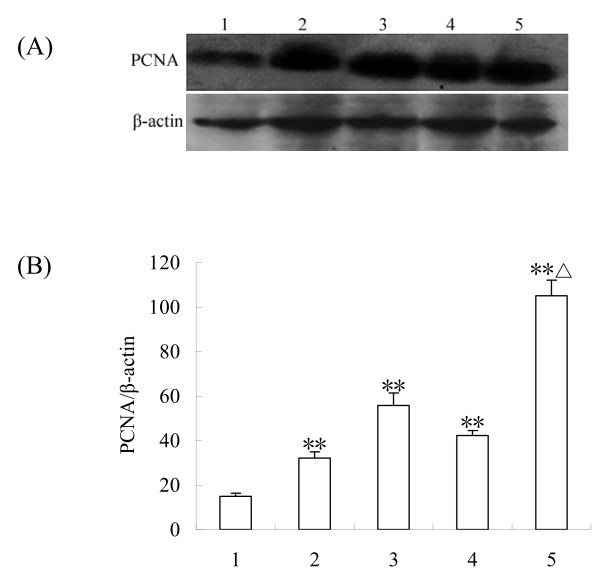
**Western blotting detection of PCNA protein in the ovaries**. (A), The expressions of. PCNA protein by western blot; (B), Semiquantitative analysis of the western-blot result. (1), Control (ovary of 2-day-old rat); (2), Ovary of 2-day-old rat cultured for 4 days; (3), Ovary of 2-day-old rat cultured for 8 days; (4), Ovary of 2-day-old rat cultured for 4 days with 50 ng/ml EGF; (5), Ovary of 2-day-old rat cultured for 8 days with 50 ng/ml EGF. Data are presented as means ± SEM (n = 3). **, P < 0.01 vs control group; Δ, P < 0.05 vs group 3.

To identify the growth of primordial follicle, the expression of proliferating cell nuclear antigen (PCNA) protein was detected by western blotting analysis. PCNA was expressed in the rat ovary, with a positive band in most of the samples. Differences in the intensity of the band from different group of ovaries were observed, depending on the cultured days. Our data indicated that PCNA protein levels increased with the cultured days, and EGF further enhanced PCNA protein levels by promoting primordial follicle development (Fig. 3).

### Effect of c-erbB_2 _siRNA on primordial follicle development

**Figure 4:**
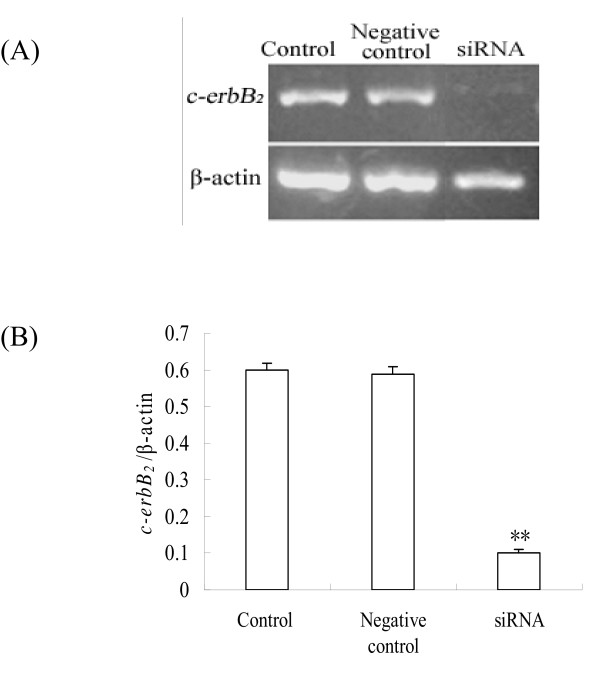
**Effect of *c-erbB***_***2***_** siRNA on *c-erbB***_***2***_** mRNA expression**. (A), Expression of *c-erbB*_*2 *_mRNA (322 bp) measured by RT-PCR in cultured ovaries after *c-erbB*_*2 *_siRNA transfection. (B), Semiquantitative analysis of the RT-PCR results. Control is the group without transfection; Negetive control is the group transfected with negative control siRNA. Data are presented as means ± SEM (n = 3). **, P < 0.01.

**Figure 5:**
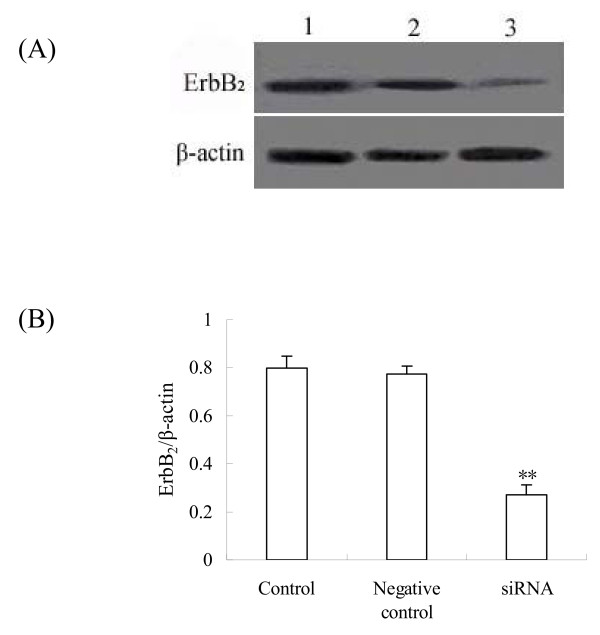
**Effect of *c-erbB***_***2***_** siRNA on ErbB**_**2 **_**protein expression**. (A), Expression of *c-erbB*_*2 *_protein in cultured ovaries after siRNA transfection by Western blot. (B), Semiquantitative analysis of the western-blot result. Control is the group without transfection; Negetive control is the group transfected with negative control siRNA. Data are presented as means ± SEM (n = 3). **, P < 0.01 vs control group.

To clarify whether *c-erbB*_*2 *_pathway was involved in initiation of growth of primordial follicle, we synthesized in vitro three siRNAs targeting the *c-erbB*_*2 *_mRNA and transferred them into the newborn rats' ovary to examine the effect of *c-erbB*_*2 *_on primordial follicle development. The siRNA with maximal effect was used in the present study (data not shown). The specificity of the *c-erbB*_*2 *_siRNA effect was verified by examining the levels of *c-erbB*_*2 *_mRNA in ovaries exposed to *c-erbB*_*2 *_siRNA. Although nontargeting control siRNA did not affect the basal transcript level of the gene, *c-erbB*_*2 *_siRNA specifically and appreciably knocked down the levels of *c-erbB*_*2 *_mRNA in ovaries cultured for 4 days. Meanwhile, ErbB_2 _protein expression was also reduced (Fig. 4 and 5).

**Figure 6:**
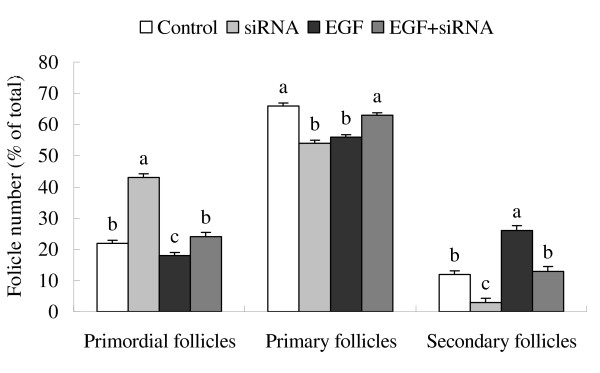
**Effect of *c-erbB***_***2***_** siRNA on the initiation of primordial follicle growth**. (A), Ovaries were cultured for 8 days with treatments: A, control; B, 0.1 mmol/L siRNA targeted *c-erbB*_*2*_; C, 50 ng/ml EGF; D, 0.1 mmol/L siRNA+50 ng/ml EGF. (B), The number of follicles was counted in serial cross-sections. Percentage of the number of each category over the total number was plotted. Data are presented as means ± SEM (n = 5). Bars with different superscripts are statistically different (P < 0.01).

A primordial follicle is composed of an oocyte surrounded by flattened pregranulosa cells, and can initiate the growth spontaneously when cultured in vitro. Statistical analysis revealed that, compared with the control (cultured for 8 days without treatment), siRNA treatment significantly inhibited the growth of primordial follicle and reduced the percentage of secondary follicles. The highest percentage of secondary follicles was observed after 8 day culture with 50 ng/ml EGF. However, EGF-stimulated increase of secondary follicles was obviously inhibited by concurrent treatment with *c-erbB*_*2 *_siRNA (Fig. 6). These data indicate that *c-erbB*_*2 *_siRNA can block spontaneous and EGF-induced activation of primordial follicles by suppressing the expression of *c-erbB*_*2*_.

### Expressions of ErbB_2_, p-ERK and p-PKC protein after c-erbB_2 _siRNA transfection

**Figure 7:**
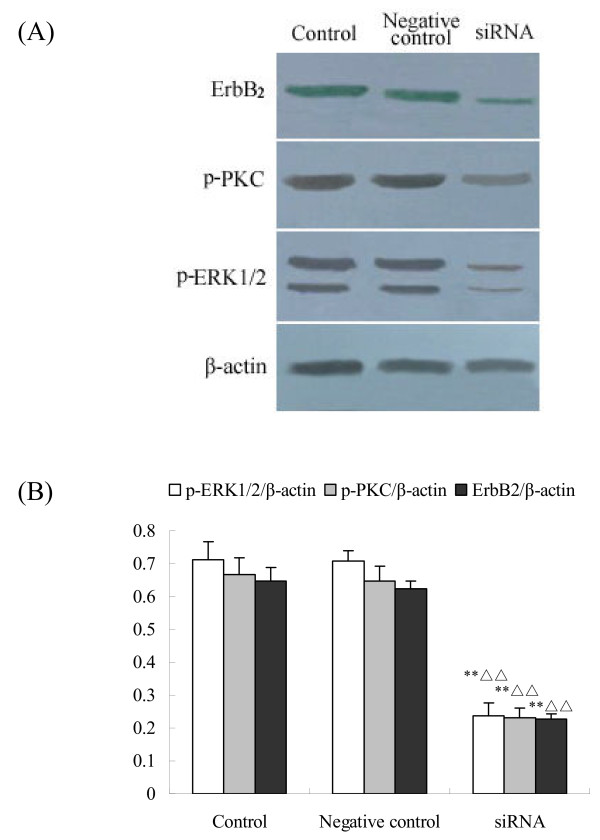
**Effect of *c-erbB***_***2***_** siRNA on the expressions of ErbB**_**2**_**, p-ERK and p-PKC protein**. (A), The expressions of ErbB_2_, p-ERK and p-PKC protein in the ovaries by western blot; (B), Semiquantitative analysis of the western-blot result. Control is the group without transfection; Negetive control is the group transfected with negative control siRNA. Data are presented as means ± SEM (n = 3). **, P < 0.01 vs control group; ΔΔs, P < 0.01 vs negative control.

To investigate the signal pathway of *c-erbB*_*2*_, siRNA was transfected into the cultured neonatal rat ovaries in vitro by liposome. After 8 day culture, western blot analysis was performed to measure the expressions of ErbB_2_, p-ERK and p-PKC protein after *c-erbB*_*2 *_siRNA transfection. As shown in Fig. 7, the expression of ErbB_2_, p-ERK and p-PKC protein were remarkably inhibited by *c-erbB*_*2 *_siRNA, compared with the control.

### Effect of PD98059 and calphostin on primordial follicle development

**Figure 8:**
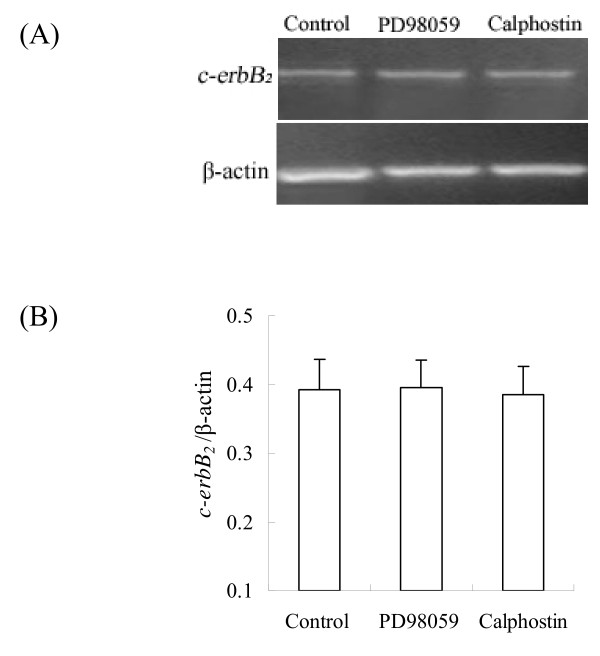
**Effect of MAPK inhibitor PD98059 and PKC inhibitor calphostin on the expression of *c-erbB***_***2***_** mRNA**. (A), The expression of *c-erbB*_*2 *_mRNA (322 bp) in the ovaries by RT-PCR; (B), Semiquantitative analysis of the RT-PCR results. Data are presented as means ± SEM (n = 3).

**Figure 9:**
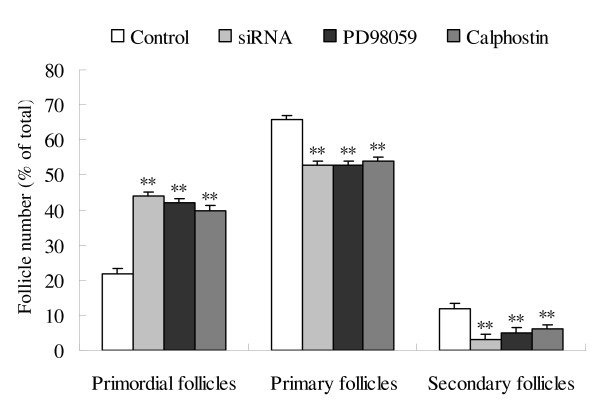
**Effect of *c-erbB***_***2***_** siRNA, PD98059 and calphostin on the initiation of primordial follicle growth**. (A), Ovaries were cultured for 8 days with treatments: A, control; B, 0.1 mmol/L siRNA; C, 5 × 10^-2 ^mmol/L PD98059; D, 5 × 10^-4 ^mmol/L calphostin. (B), The number of follicles was counted in serial cross-sections. Percentage of the number of each category over the total number was plotted. Data are presented as means ± SEM (n = 5) **, P < 0.01 vs control.

The expression of *c-erbB*_*2 *_mRNA were examined by RT-PCR. The results showed that PD98059 and calphostin did not significantly affect the expression of *c-erbB*_*2 *_mRNA (Fig. 8). Compared with the control, *c-erbB*_*2 *_siRNA, PD98059 and calphostin significantly inhibited the primordial to primary follicle transition of primordial follicles after 8 days culture. The number of primordial follicles was markedly increased and the number of primary follicles and secondary follicles was obviously decreased (Fig. 9). These data suggest that MAPK and PKC pathways are involved in initiation of growth of rat primordial follicles.

## Discussion

Follicles form when some of primordial germ cells are enveloped by a single layer of flattened pre-granulosa cells (pre-GC). When some follicles leave the resting pool and start the initiation of follicular growth (follicle activation), the granulosa cells (GC) become cuboidal and begin to express markers of cell proliferation, such as PCNA. EGF is essential to initiate growth of primordial follicles [17,26]. Our previous results suggested that *c-erbB*_*2 *_played an important role in the regulation of functions of granulosa cells and maturation of oocytes [24,27]. Interestingly, *c-erbB*_*2 *_also mediates spermatogonial proliferation in newt testis [28]. Therefore, we hypothesized that EGF might stimulate the initiation of primordial follicles growth via the *c-erbB*_*2 *_pathway.

In the present study, we examined the expression of *c-erbB*_*2 *_during primordial folliculogenesis and investigated the influence of EGF on *c-erbB*_*2 *_expression as well as the effects of *c-erbB*_*2 *_down-regulation on the initiation of primordial follicle growth and on the activating role of EGF. ErbB_2 _protein plays the role of epidermal growth factor receptor (EGFR) and hasn't a specific ligand. ErbB receptor has distinct signaling properties depending on its dimerization. ErbB_2_, the preferred heterodimerization partner of all ErbB receptors, is a mediator of lateral signaling [29]. We investigated the relation between *c-erbB*_*2 *_and MAPK or PKC signaling pathways during primordial folliculogenesis. Our study revealed that expression of *c-erbB*_*2 *_mRNA was present in oocytes of primordial follicles, and also appeared in cuboidal granulosa cells after initiation of follicular growth. The expression of *c-erbB*_*2 *_mRNA increased in proliferated multilayer granulosa cells after prolonged culture. EGF promoted PCNA protein expression and follicular growth by initiating primordial follicle development. In addition, EGF promoted the expression of *c-erbB*_*2 *_mRNA. Therefore, we conjecture that EGF and *c-erbB*_*2 *_might be involved in the onset of primordial follicle development.

To further understand the action of *c-erbB*_*2 *_during primordial folliculogenesis, we used the synthetic siRNA for *c-erbB*_*2 *_to transfect ovarian cells in organ culture. We observed the condition of the growth initiation of primordial follicles through inducing *c-erbB*_*2 *_gene silencing. In the current experiment, most of the primordial follicles in the control group developed to the primary follicles, whereas the number of primary follicles and secondary follicles was significantly decreased by *c-erbB*_*2 *_siRNA. Furthermore, *c-erbB*_*2 *_siRNA blocked the promoting effect of EGF on the initiation of primordial follicle growth. ErbB_2_, an orphan receptor tyrosine kinase, which can dimerize with other ligand-activated members of the EGF receptor family, might be a selecting marker for initiation of follicular growth. We observed that *c-erbB*_*2 *_siRNA inhibited the expression of ErbB_2 _protein. These results suggest that *c-erbB*_*2 *_plays an important role on the initiation of primordial follicle growth and mediates the regulating role of EGF as a key signal molecule.

**Figure 10:**
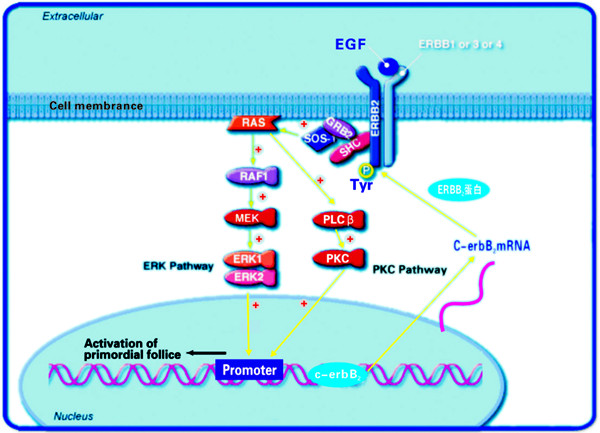
**EGF signal transduction figure**.

A variety of signaling pathways, including the MAPK and PKC regulating systems, are involved in the initiation of the growth of primordial follicles [30,31]. Phosphorylated MAPK (active) exists in some proliferating granulosa cells, and the activity of MAPK constantly increases during he process of oogonium proliferation [32]. The PKC family has been implicated in various functional responses on the regulation of cell development including cell growth, cell cycle progression, cell survival, apoptosis and cell differentiation. As a potent and selective inhibitor of MAP kinase kinase (MEK), PD-98059 blocks activation of MEK binding to the ATP site of dephosphorylation MEK, thereby inhibites phosphorylation and activation of MAP kinase1, 2 (ERK1/2) [33]. Calphostin C, a potent inhibitor of protein kinase C, inhibits phorbol dibutyrate binding to PKC [34]. In this study, both PD98059 and calphostin significantly inhibited the development of primordial follicles, suggesting that MAPK and PKC signal pathways are involved in the initiation of primordial follicle growth. However, the upstream and downstream relationship between MAPK or PKC and *c-erbB*_*2 *_is still unclear during primordial folliculogenesis. Therefore, we investigated the MAPK and PKC pathways as possible mediators for the expression of *c-erbB*_*2 *_using calphostin and PD98059. Both inhibitors did not change the expression of *c-erbB*_*2 *_mRNA, while the expression levels of PKC and MAPK protein were significantly decreased by *c-erbB*_*2 *_siRNA transfection in primordial follicles. These results indicated that *c-erbB*_*2 *_might be an upstream activator of MAPK and PKC, which regulated the initiation of primordial follicle growth at least in part via the activation of MAPK and PKC signal pathways (Fig.10).

A complex signal network system composed of a variety of autocrine, paracrine and endocrine factors regulates the growth of primordial follicles via intercellular communications, and it has been demonstrated that the growth of primordial follicles was associated with precise spatiotemporal expression of multiple genes and interactions between these genes [3,4,16,17,26,35,36]. The signaling pathways, such as PI3K and mTORC1 pathways, regulate the activation of primordial follicles and the early development of ovarian follicles [37,38]. However, the exact mechanism by which the various factors regulate the growth of primordial follicles has not yet been fully understood. *c-erbB*_*2 *_might have roles in the growth of primordial follicles beyond that of mediating EGF signaling. It also might regulate proliferation of granulosa cells and cumulus cells, which have close signaling communication with oocytes, to govern initiation of follicular growth, development, and steroidogenesis. Futher research of *c-erbB*_*2 *_functions may provide novel information for understanding the mechanism of the follicular initiation and development.

## Conclusions

In conclusion, we showed that EGF promoted the initiation of primordial follicle development and the expression of *c-erbB*_*2 *_in ovaries, whereas the promoting effect of EGF was blocked by *c-erbB*_*2 *_siRNA transfection. In addition, the initiation of primordial follicle growth was inhibited by MAPK or PKC inhibition. The expression of ErbB_2_, p-ERK and p-PKC protein and primordial follicle development were inhibited by *c-erbB*_*2 *_siRNA transfection. These results indicated that *c-erbB*_*2 *_played an important role in primordial follicle initiation and development and the effect of *c-erbB*_*2 *_might be mediated by a mechanism involving the PKC and MAPK pathways.

## Competing interests

The authors declare that they have no competing interests.

## Authors' contributions

ZLP carried out all the experiments. ZDL, HJ, XLQ, XAX, DXY and TDF performed statistical analysis and drafted the paper. ZYH designed the study and amended the paper. All authors read and approved the final manuscript.
